# Stability of UV/ozone-treated thermoplastics under different storage conditions for microfluidic analytical devices†
†Electronic supplementary information (ESI) available. See DOI: 10.1039/c7ra07435b


**DOI:** 10.1039/c7ra07435b

**Published:** 2017-07-28

**Authors:** Tung-Yi Lin, Trey T. Pfeiffer, Peter B. Lillehoj

**Affiliations:** a Department of Mechanical Engineering, Michigan State University, East Lansing, MI, USA. Email: lillehoj@egr.msu.edu; b Department of Biomedical Engineering, Michigan State University, East Lansing, MI, USA

## Abstract

Thermoplastics are becoming a popular material for fabricating microfluidic devices and there is an increasing need for robust surface modification strategies. UV/ozone (UVO) treatment is a simple and effective method for making plastic surfaces more hydrophilic. Prior reports on the stability of UVO-treated plastics are limited to four weeks, which is not sufficient for applications requiring long-term storage. Here, we present new findings on the long-term stability of UVO-treated plastics for up to 16 weeks and show that the storage condition has a significant impact on the surface stability. Static contact angle measurements, X-ray photoelectron spectroscopy (XPS) and atomic force microscopy (AFM) were performed on UVO-treated cyclic olefin copolymer (COC), polycarbonate (PC) and poly(methyl methacrylate) (PMMA) stored in air, dehumidified and vacuum conditions. We found that the hydrophobic recovery of UVO-treated COC and PC can be inhibited by storing them in dehumidified or vacuum conditions, whereas the stability of PMMA is not significantly influenced by the storage condition. Protein adsorption studies were carried out and showed that there is a significant reduction in the amount of protein adsorption on UVO-treated plastics compared with untreated plastics. Lastly, UVO-treated PMMA microchannels were fabricated and used for capillary-driven flow, which revealed that longer treatment durations generate faster flow rates. These collective results offer new insights into the utility of UVO-treated plastics for microfluidic analytical applications.

## Abstract 2

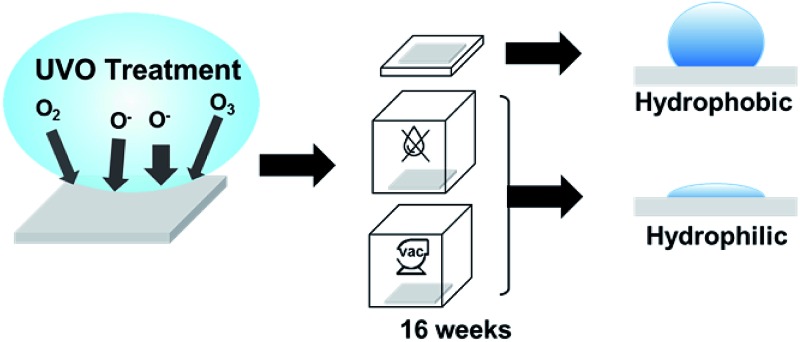
Hydrophobic recovery of UVO-treated plastics can be inhibited by storing them in dehumidified or vacuum conditions.

## Introduction

Microfluidics is becoming a mainstream technology in biomedical research where it has already made significant advances in molecular biology, analytical chemistry and drug discovery. Traditionally, microfluidic devices are fabricated from poly(dimethylsiloxane) (PDMS) and glass using well-established methods, such as soft lithography.^1^ While PDMS offers many attractive properties for biomedical applications, it suffers from several drawbacks, including limited compatibility with some chemicals,^2^ adsorption of hydrophobic small molecules^3^ and limited stability.^4^ Additionally, the fabrication of PDMS microfluidic devices involves multiple processes and is not well suited for high-volume production. Thermoplastics, on the other hand, exhibit good hydrolytic stability,^5^ and are amenable to both low- and high-volume manufacturing processes.^6^,^7^ Plastics are also compatible with many of the same surface treatments as PDMS and glass, enhancing their versatility for applications requiring surface functionalization.^8^,^9^ For these reasons, plastics are becoming more commonly used to fabricate microfluidic devices for various biomedical applications, such as DNA analysis,^10^ immunosensing,^11^,^12^ cell studies/cell culture^13^,^14^ and tissue engineering.^15^

Most commercially available plastics are hydrophobic in their native form, which can result in the adsorption of molecules and influence the accuracy of analytical measurements.^8^ Various methods have been reported to make plastic surfaces more hydrophilic, such as chemical grafting,^16^ oxygen plasma etching^17^ and UV/ozone (UVO) exposure.^18^ Of these techniques, chemical grafting offers broad versatility in regards to surface chemistry, but can involve long and complicated fabrication processes and/or require the use of expensive equipment. Alternatively, surface oxidation *via* exposure to an oxygen plasma is a simple method to enhance the surface hydrophilicity. While effective, the oxidized surface undergoes hydrophobic recovery within a couple of hours.^19^ Plastic surfaces can also be oxidized by UVO treatment which results in minimal hydrophobic recovery for up to 4 weeks.^20^ While these results are useful for some applications, further work is needed to evaluate the long-term stability of UVO-treated plastics and its utility for microfluidic analytical applications.

In this work, we studied the stability of UVO-treated plastics under different storage conditions for 16 weeks. We focused our efforts on cyclic olefin copolymer (COC), polycarbonate (PC) and poly(methyl methacrylate) (PMMA), which are among the most common plastics for microfluidic devices due to their excellent biocompatibility, high optical transparency and suitability for mass production.^21^ Surface characterization of untreated and UVO-treated plastics was performed using X-ray photoelectron spectroscopy (XPS), atomic force microscopy (AFM) and contact angle measurements to assess surface wettability. We also investigated the influence of UVO treatment on protein adsorption owing to its importance for analytical applications. Lastly, we studied the effectiveness of UVO treatment on PMMA for generating capillary-driven flow in microfluidic devices.

## Materials and methods

### UVO treatment and sample storage

PC and PMMA were purchased from McMaster-Carr (Elmhurst, IL) and COC was purchased from Zeon Chemicals (Louisville, KY). For XPS and contact angle measurements, COC and PMMA were cut into 1 cm × 2 cm pieces using a CO_2_ laser cutter (Universal Laser Systems, Scottsdale, AZ) and PC was cut using a band saw. UVO treatment was performed using a UVO cleaner (Novascan Technologies, Ames, IA). For stability studies, samples were treated for 20, 40, 60 or 80 min at room temperature and stored in air, a dehumidified chamber (∼20% relative humidity) or vacuum-sealed plastic bags (Weston vacuum sealer, Strongsville, OH).

### Contact angle measurements

Static contact angle measurements were carried out using a VCA-2000 video contact angle analysis system (AST Products Inc., Billerica, MA). 0.5 μL droplets of distilled water were dispensed onto the samples using a pipette. Contact angle data was acquired at ambient conditions from three different samples at a minimum of three locations per sample. Each data point is plotted as the mean of eight separate measurements with outliers removed. New samples were used for each set of measurements.

### XPS analysis

XPS spectra were obtained using a Perkin Elmer Phi 5400 ESCA system (Physical Electronics, Chanhassen, MN) at pressures between 10^–9^ and 10^–8^ torr, pass energy of 29.35 eV, and a 45° take-off angle. Elemental composition was calculated from the relative intensities of the C1s and O1s peak areas obtained from the survey spectral after subtraction of a linear background.

### Atomic force microscopy

AFM scans were performed under tapping mode in air using a Cypher atomic force microscope (Asylum Research, Goleta, CA). Samples were imaged using a force constant of 0.2 N m^–1^, scan rate of 2.44 Hz and scan size of 4 μm × 4 μm. Images were processed and analyzed for Root Mean Square (RMS) surface roughness using Igor Pro software (WaveMetrics, Lake Oswego, OR).

### Biochemicals and reagents

Horseradish peroxidase (HRP)-labeled anti-*Pf*HRP2 antibody was obtained from Immunology Consultants Laboratory, Inc (Lake Oswego, OR) and diluted to 10 μg mL^–1^ in phosphate buffered saline (PBS) (Fisher Scientific, Pittsburgh, PA). 3′,3′,5′,5′ tetramethylbenzidine substrate (TMB/H_2_O_2_) was obtained from Neogen (Lexington, KY), and deionized (DI) water (18.3 MΩ cm) was generated using a Thermo Scientific Smart2Pure water purification system.

### Protein adsorption measurements

**Fig. 1:**
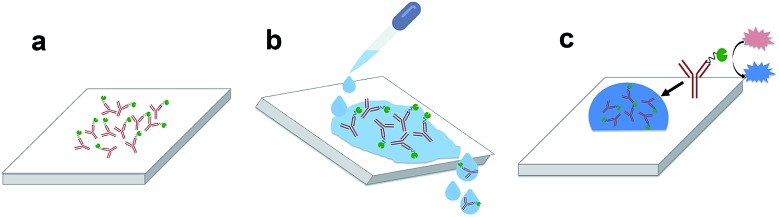
Schematic showing the process for protein adsorption measurements. (a) Enzyme-labeled antibodies adsorbed on the surface after 30 min of incubation. (b) Samples are rinsed with PBS and DI water to remove unabsorbed proteins. (c) TMB/H_2_O_2_ is dispensed on the surface and incubated for 1 min followed by absorbance measurements at 650 nm.

PMMA, PC and COC were cut into 2 cm × 2 cm pieces and a 7 mm-diameter circular well was formed by attaching an adhesive-backed plastic stencil on the surface. Plastic pieces were exposed to UVO for 20, 40, 60 or 80 min followed by removal of the stencil. 80 μL of 1 μg mL^–1^ antibody solution was dispensed onto each piece and incubated for 30 min, rinsed using PBS and DI water, and dried under a stream of purified N_2_. This process resulted in the adsorption of enzyme-labeled antibodies on the surface (Fig. 1a). Pieces were then rinsed using PBS and DI water to remove unabsorbed proteins (Fig. 1b) and dried using N_2_. 80 μL of TMB/H_2_O_2_ was dispensed onto each sample and incubated for 1 min (Fig. 1c) followed by absorbance measurements at 650 nm using a UV-visible spectrometer (Shimadzu, Kyoto, Japan).

### Microchannel fabrication

**Fig. 2:**
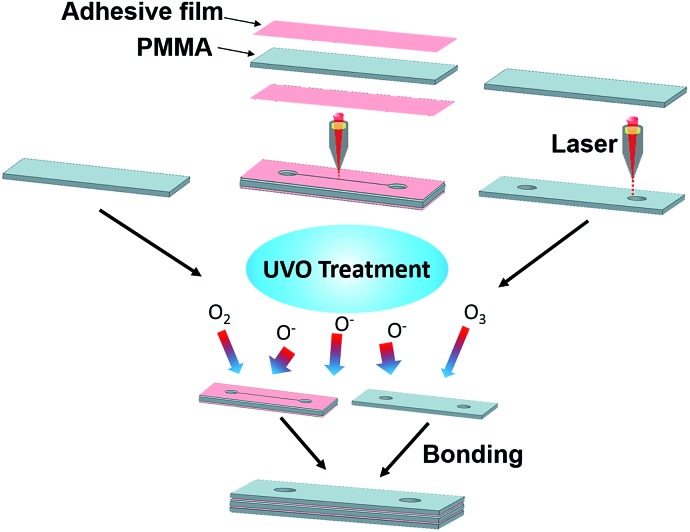
Fabrication process flow for UVO-treated PMMA microchannels. Each device is comprised of three pieces of PMMA bonded together using adhesive film.

Microfluidic devices were fabricated by etching microfluidic features in PMMA using a CO_2_ laser cutter, followed by UVO treatment and bonding using double-sided adhesive film (Adhesive Research, Glen Rock, PA). A schematic of the fabrication process is shown in Fig. 2. The width, height and length of the channel are 150 μm, 1.87 mm and 30 mm, respectively, and the diameter of the inlet and outlet is 2 mm.

## Results and discussion

### Surface chemistry and morphology of UVO-treated plastics

UVO treatment of plastic results in the generation of an oxide layer on the surface, rendering it more hydrophilic.^20^ Organic contaminants on the surface are removed *via* non-destructive atomic layer etching by 185 nm and 254 nm ultraviolet light. In the presence of oxygen, 185 nm light generates ozone while 254 nm light simultaneously excites organic molecules on the surface. The 254 nm light also generates free radicals on the surface that react with oxygen, generating more oxygen-containing species.^22^ The generation of these oxygen-containing species increases the surface free energy and lowers the hydrophobicity. To study the change in the surface chemistry as a result of UVO treatment, XPS spectra of plastics exposed to UVO at varying durations were obtained and used to calculate the oxygen/carbon (O/C) ratio on the surface. As shown in Table 1, all three UVO-treated plastics exhibit significantly higher O/C ratios compared with untreated plastics, which is due to the presence of additional oxygen-containing functional groups, and indicative of the formation of an oxide layer. In addition, there is a positive correlation between the O/C ratio and treatment duration, where longer UVO exposure results in a higher O/C ratio since more oxygen-containing species are generated over time. The generation of oxygen species occurs rapidly within the first 20 min and gradually lessens with longer exposure times as the surface becomes saturated. After a certain point (∼60 min), the oxygen content on the surface reaches a plateau, as indicated by a negligible (<5%) change in the O/C ratio compared with those of plastics with longer (80 min) UVO exposure.

**Table 1:** XPS analysis of the O/C ratio on the surface of UVO-treated and untreated plastics immediately following UVO exposure

		PMMA	COC	PC
Untreated		0.34 ± 0.05	0.12 ± 0.02	0.19 ± 0.02
UVO-treated	20 min	0.48 ± 0.07	0.42 ± 0.10	0.47 ± 0.12
	40 min	0.54 ± 0.09	0.56 ± 0.06	0.59 ± 0.04
	60 min	0.53 ± 0.02	0.54 ± 0.07	0.55 ± 0.04
	80 min	0.56 ± 0.08	0.55 ± 0.05	0.56 ± 0.04

AFM scans (Fig. S1 in ESI†) and surface roughness measurements (Table S1 in ESI†) of UVO-treated and untreated samples were also obtained to briefly study the influence of UVO treatment on surface morphology. These results show that UVO-treated plastics exhibit smoother surface profiles and 2–7× smaller roughness values compared with untreated plastics. The smoother surfaces of UVO-treated plastics is due to the removal of organic contaminants *via* non-destructive etching from the UVO treatment process.

### Influence of UVO treatment on surface wettability

**Fig. 3:**
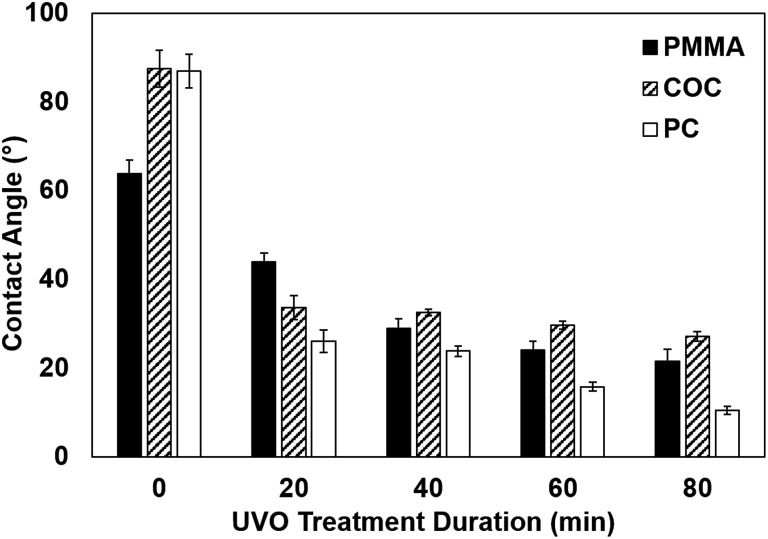
Contact angles of PMMA, COC and PC with varying durations of UVO treatment. Each bar represents the mean ± standard deviation (SD) of eight separate measurements.

We assessed the surface wettability of UVO-treated plastics by performing static contact angle measurements of PMMA, COC and PC treated for varying durations (Fig. 3). COC and PC exhibit substantial (60.6% ± 2.5% and 70.1% ± 3.2%, respectively) reductions in contact angle with 20 min of UVO exposure, whereas PMMA exhibits a moderate (31.0% ± 2.9%) reduction. However, with 40 min of UVO treatment, the contact angle of PMMA is reduced by 54.7 ± 0.7%, which is similar to COC and PC. With longer treatment times, the contact angles of COC and PMMA reach steady state at 60 min and 80 min for PC, which is indicative of the surface becoming saturated with oxygen-containing species. Among all three plastics, PC exhibits the lowest contact angle of 10.5° ± 0.6° after 80 min of UVO treatment, resulting in a superhydrophilic surface.^23^ For PC and PMMA, there is an observable correlation between the treatment duration and surface wettability, where longer times result in lower contact angles. In contrast, UVO treatment times >20 min have a negligible impact on reducing the contact angle of COC, which is consistent with findings reported by Bhattacharyya *et al.*^18^ These results indicate that 20 min of UVO treatment is sufficient in generating a hydrophilic surface on all three plastics, where a more substantial enhancement in wettability can be obtained for PMMA and PC with longer treatment durations.

### Analysis of UVO-treated plastics under different storage conditions

**Fig. 4:**
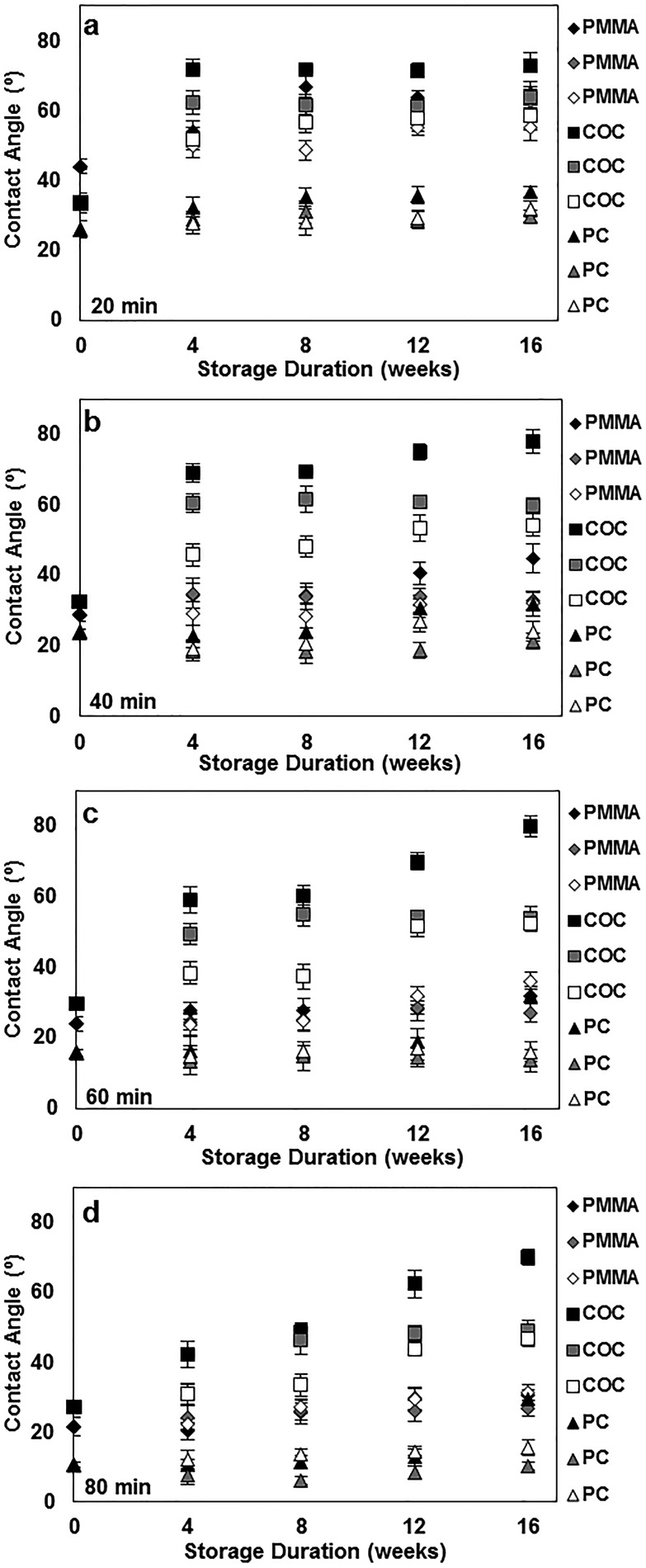
Long-term contact angle measurements of PMMA, COC and PC treated for 20 min (a), 40 min (b), 60 min (c) and 80 min (d) under various storage conditions. Black, grey and white markers correspond to storage in air, dehumidified and vacuum conditions, respectively. Each data point represents the mean ± SD of eight separate measurements.

To better understand the long-term stability of UVO-treated plastics, we performed contact angle measurements of plastics treated with UVO for 20 min (Fig. 4a), 40 min (Fig. 4b), 60 min (Fig. 4c) and 80 min (Fig. 4d) over the course of 16 weeks. For this study, plastics were stored under different conditions (air, dehumidified and vacuum) to determine its influence on hydrophobic recovery. Plastic samples stored in air, dehumidified and vacuum conditions are represented as black, grey and white markers, respectively, in Fig. 4. When stored in air, COC experiences substantial hydrophobic recovery within 4 weeks, while PC and PMMA experience moderate hydrophobic recovery. In general, this trend is consistent for all four treatment times. When stored in air for >4 weeks, the degree of hydrophobic recovery increases slightly for all three plastics, with the exception of COC treated for >40 min which steadily increases over time. More importantly, these measurements show that the influence of the storage condition on the hydrophobic recovery is different for each type of plastic. For PMMA, the storage condition has a minimal impact on the surface stability for the duration of the study, particularly samples treated >40 min. This trend is similar for PC, however after 12 weeks, there is a noticeable increase in the contact angle for samples stored in air. In contrast, the storage condition has a significant impact on the surface stability of COC, where the hydrophobic recovery is dramatically reduced by storing samples in vacuum. These results indicate that the surface stability can be enhanced by longer UVO exposure and storage in either a dehumidified or vacuum environment.

XPS spectra of UVO-treated (80 min) plastics stored for 16 weeks under different conditions were also obtained and used to calculate O/C ratios, which are presented in Table 2. Compared with freshly treated samples (Table 1), the O/C ratios for stored samples are lower, indicating a reduction in the concentration of oxygen-containing species on the surface due to hydrophobic recovery. UVO-treated plastics stored in air exhibit substantially smaller O/C ratios by 50% compared with those freshly treated. In contrast, UVO-treated plastics stored in dehumidified and vacuum conditions exhibit smaller (∼10% and ∼5%, respectively) reductions in the O/C ratio. These results are consistent with our long-term contact angle measurements (Fig. 4d) which show that hydrophobic recovery is generally more pronounced when stored in air and significantly inhibited when stored in dehumidified or vacuum conditions. The slightly higher O/C ratios exhibited by plastics stored in a dehumidified environment compared with in vacuum is likely due to the adsorption of water molecules on the UVO-treated surfaces, which react with the oxide layer subsequently reducing the oxygen content on the surface. Therefore, the oxide layer of UVO-treated plastics can be maintained by isolating them from moisture-rich environments, thereby preserving the surface hydrophilicity.

**Table 2:** XPS analysis of the O/C ratio on the surface of UVO-treated plastics after 16 weeks under different storage conditions

	PMMA	COC	PC
Air	0.27	0.31	0.34
Dehumidified	0.41	0.38	0.38
Vacuum	0.34	0.34	0.36

AFM scans (Fig. S2 in ESI†) and surface roughness measurements (Table S1 in ESI†) of UVO-treated plastics stored in air and vacuum for 16 weeks were obtained to briefly study the change in surface morphology due to the storage condition. These results show that plastics stored in air exhibit rougher surface profiles and 2–5× larger roughness values compared with plastics stored in vacuum. The increased surface roughness of samples stored in air is mainly due to the adsorption of organic contaminants from air,^24^ which contributes to its faster hydrophobic recovery. We also briefly studied the influence of storage temperature on surface stability by measuring the contact angle of UVO-treated (80 min) plastics stored in air at room temperature and 50 °C after 1, 3 and 7 days of storage (Fig. S3 in ESI†). These measurements show that PMMA and COC stored at 50 °C experience hydrophobic recovery more rapidly compared with samples stored at room temperature. In contrast, PC stored at 50 °C exhibits only a slight increase in hydrophobic recovery after 7 days of storage. These results are consistent with prior studies which report that elevated storage temperature results in faster hydrophobic recovery of plasma-treated surfaces stored in air.^19^,^24^

### Protein adsorption on UVO-treated plastics

**Fig. 5:**
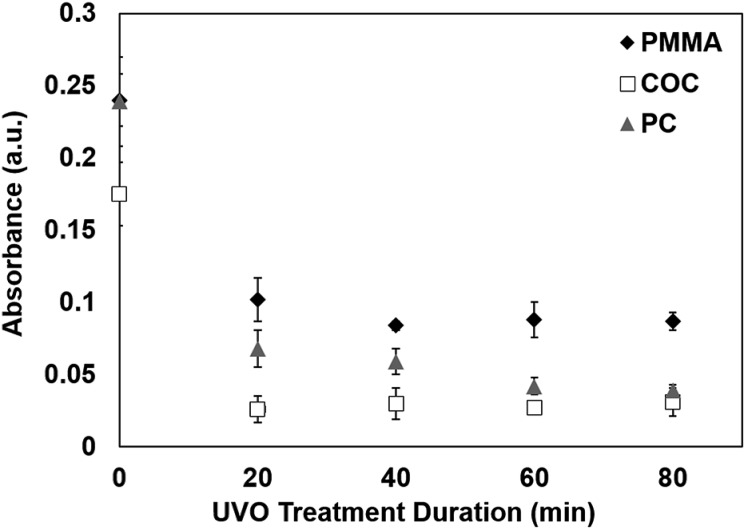
Absorbance values of TMB/H_2_O_2_ on plastics exposed to UVO at varying durations. Each data point represents the mean ± SD of three separate measurements obtained using new samples.

The surface hydrophilicity plays an important role in protein adsorption.^25^ To investigate the effect of UVO treatment on protein adsorption, we used a colorimetric detection scheme based on an enzymatic reaction between an HRP-labeled antibody and a chromogenic substrate. Briefly, a droplet of solution containing HRP-labeled antibody was incubated on a plastic sample for 30 min to allow the protein complex to adsorb onto the surface. The surface was rinsed using PBS and DI water to remove unabsorbed proteins, followed by the application of the substrate (TMB/H_2_O_2_). The substrate reacts with the surface-adsorbed HRP to generate a distinct blue color. The absorbance of the droplet was measured at 650 nm using a UV-visible spectrometer, which correlates with the amount of protein adsorbed on the surface. Absorbance measurements were performed on PMMA, COC and PC exposed to UVO at varying durations. As shown in Fig. 5, longer UVO treatment durations result in lower absorbance values, indicating that more hydrophilic surfaces inhibits the absorption of proteins. These findings are consistent with prior reports on the interactions between proteins and hydrophilic surfaces.^26^ Of the three plastics studied, COC exhibits the lowest absorbance values for all treatment times, indicating the least amount of protein absorption. In contrast, PMMA exhibits the highest absorbance values, even after 80 min of UVO treatment, indicating more protein adsorption which is likely due to its surface functional groups^27^ and ionic strength.^28^ These results indicate that 20 min of UVO treatment is sufficient in lowering the overall amount of protein adsorption on plastic surfaces, where more substantial reductions can be obtained on PC with longer treatment durations.

### Capillary flow in UVO-treated microfluidic devices

**Fig. 6:**
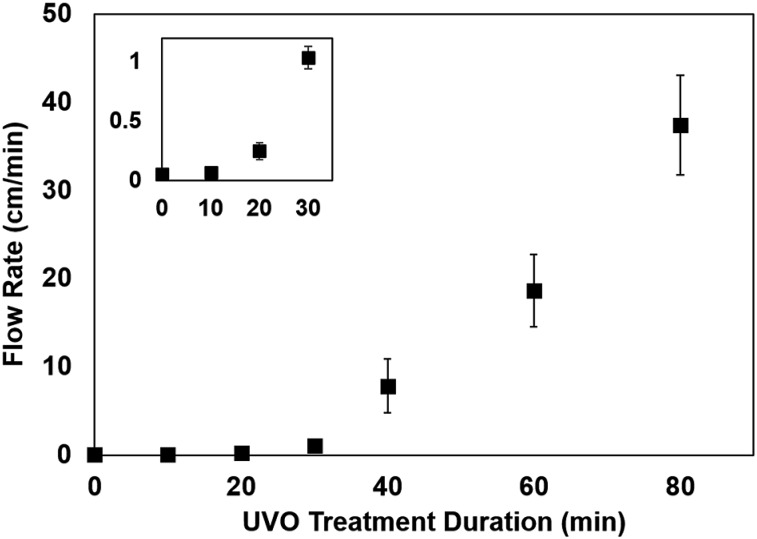
Capillary flow rate as a function of UVO treatment duration for PMMA microchannels. Inset shows magnified view of the data points at lower (<40 min) UVO exposure times. Each data point represents the mean ± SD of three measurements.

Capillary-driven flow is one of the simplest techniques for liquid transport in microfluidic devices. The capillary flow rate is highly dependent on the contact angle of the channel walls,^29^ which is associated with the surface wettability. Therefore, we briefly studied the influence of UVO treatment duration on the capillary flow rate in PMMA microchannels. Colored dye was dispensed in microchannels with varying UVO treatment durations and the resulting flow rates were measured. As shown in Fig. 6, there is a positive correlation between the UVO treatment duration and the flow rate, where longer treatment times result in faster flows. These results are consistent with our contact angle measurements (Fig. 3) which show that longer UVO treatment durations result in enhanced surface wettability. Based on this approach, microchannels with defined capillary flow rates can be achieved by simply adjusting the UVO treatment duration. Furthermore, UVO-treated microchannels can be stored for several months in dehumidified or vacuum conditions to preserve the surface stability.

## Conclusions

We have presented new findings on the surface chemistry, morphology and long-term stability of UVO-treated plastics through XPS, AFM and contact angle measurements. Specifically, we showed that UVO-treated PMMA, COC and PC experience hydrophobic recovery within 4 weeks and the rate at which it occurs is dependent on the UVO treatment duration. Furthermore, we have discovered that the hydrophobic recovery of UVO-treated COC and PC can be inhibited by storing them in dehumidified or vacuum conditions. XPS measurements revealed that plastics stored in dehumidified and vacuum conditions exhibit higher oxygen content on the surface compared with plastics stored in air. UVO-treated plastics were also used for protein adsorption measurements, which showed that UVO treatment minimizes protein adsorption and this effect is correlated with the treatment duration. Lastly, we demonstrated capillary-driven flows in UVO-treated PMMA microchannels, which revealed that the flow rate can be tuned by adjusting the treatment duration. These collective results offer new insights into the long-term stability of UVO-treated plastics, as well as their utility for microfluidic analytical applications.

## Conflict of interest

There are no conflicts of interest to declare.

## Supplementary Material

Supplementary information
