# Supplementary material for: Stability of UV/ozone-treated thermoplastics under different storage conditions for microfluidic analytical devices
Source: RSC Adv. 2017 Jul 28;7(59):37374–9. doi: 10.1039/c7ra07435b (PMC5708361; doi:10.1039/c7ra07435b)
Supplement: Supplementary file 1 [file RA-007-C7RA07435B-s001.pdf]

Electronic Supplementary Material (ESI) for RSC Advances.
This journal is © The Royal Society of Chemistry 2017

Supplementary Information

Stability of UV/Ozone-treated thermoplastics under different storage conditions for microfluidic analytical devices

Tung-Yi Lin,[a] Trey T. Pfeiffer,[a] and Peter B. Lillehoj[a, b*]

[a] Department of Mechanical Engineering, Michigan State University, East Lansing, MI USA

[b] Department of Biomedical Engineering, Michigan State University, East Lansing, MI USA

*lillehoj@egr.msu.edu, Tel: +1-517-432-2976

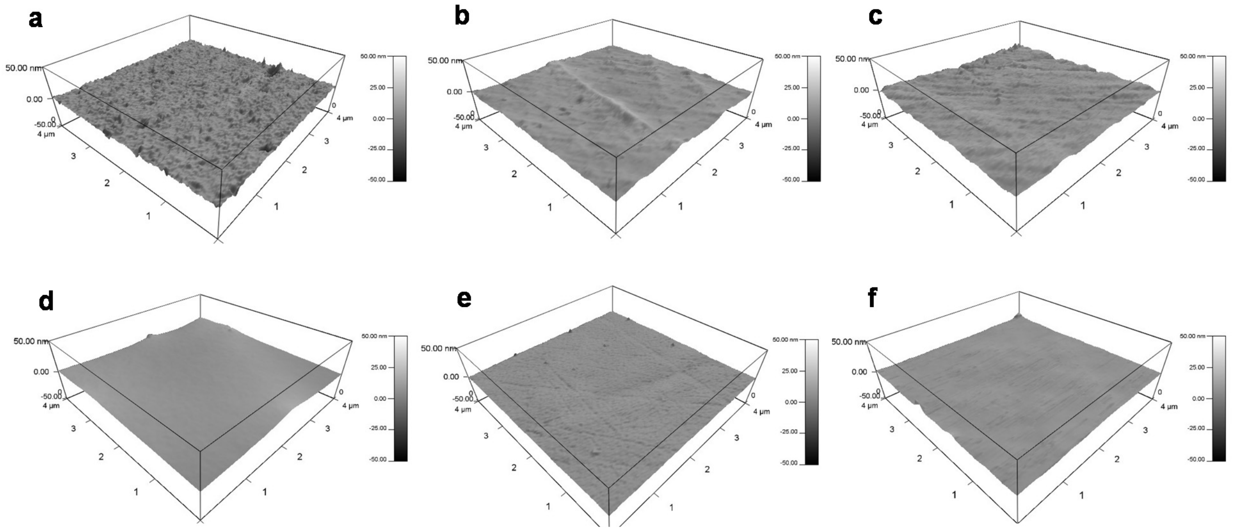

Fig. S1 AFM tapping mode images of untreated PMMA (a), COC (b), and PC (c), and UVO-treated (80 min) PMMA (d), COC (e) and PC (f). The scan size and z-scale are 4 μm × 4 μm and 50 nm, respectively.

Table S1 Surface roughness values (nm) of UVO-treated and untreated plastic samples immediately after treatment, and after 16 weeks of storage under different storage conditions

		PMMA	COC	PC
**Untreated, Non-stored**		1.99 ± 1.34	2.21 ± 1.64	1.99 ± 1.57
**UVO-treated (80 min)**	**Non-stored**	0.26 ± 0.21	0.70 ± 0.53	0.83 ± 0.62
	**Stored in air**	2.76 ± 1.91	2.93 ± 2.36	2.26 ± 1.59
	**Stored in vacuum**	0.51 ± 0.35	1.10 ± 0.70	1.13 ± 0.62

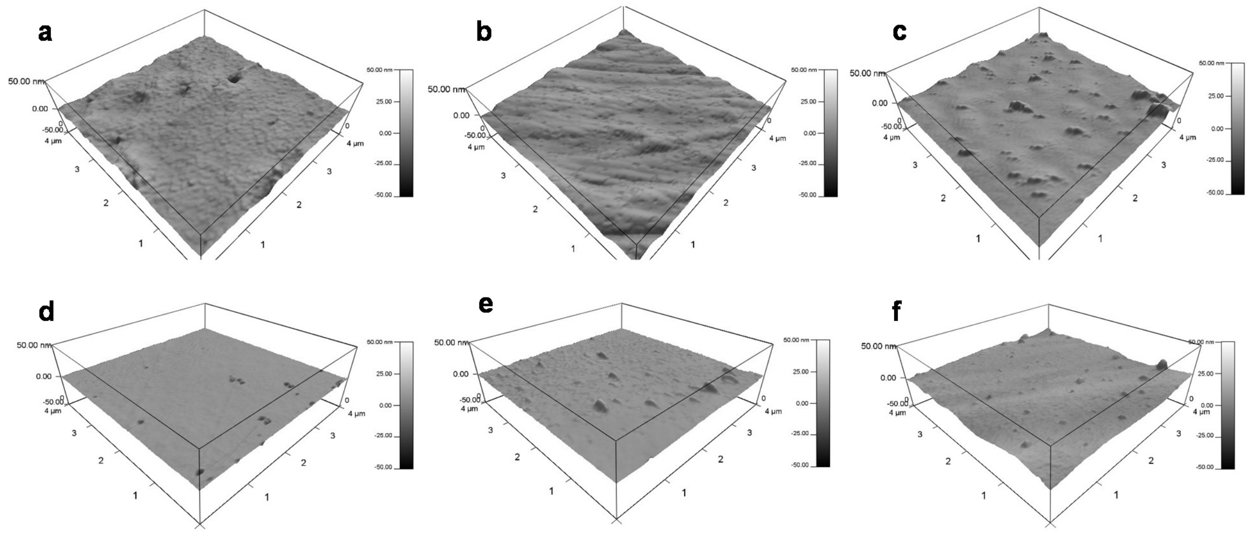

Fig. S2 AFM tapping mode images UVO-treated (80 min) PMMA (a), COC (b), and PC (c) stored in air, and PMMA (d), COC (e), and PC (f) stored in vacuum for 16 weeks. The scan size and z-scale are 4 μm × 4 μm and 50 nm, respectively.

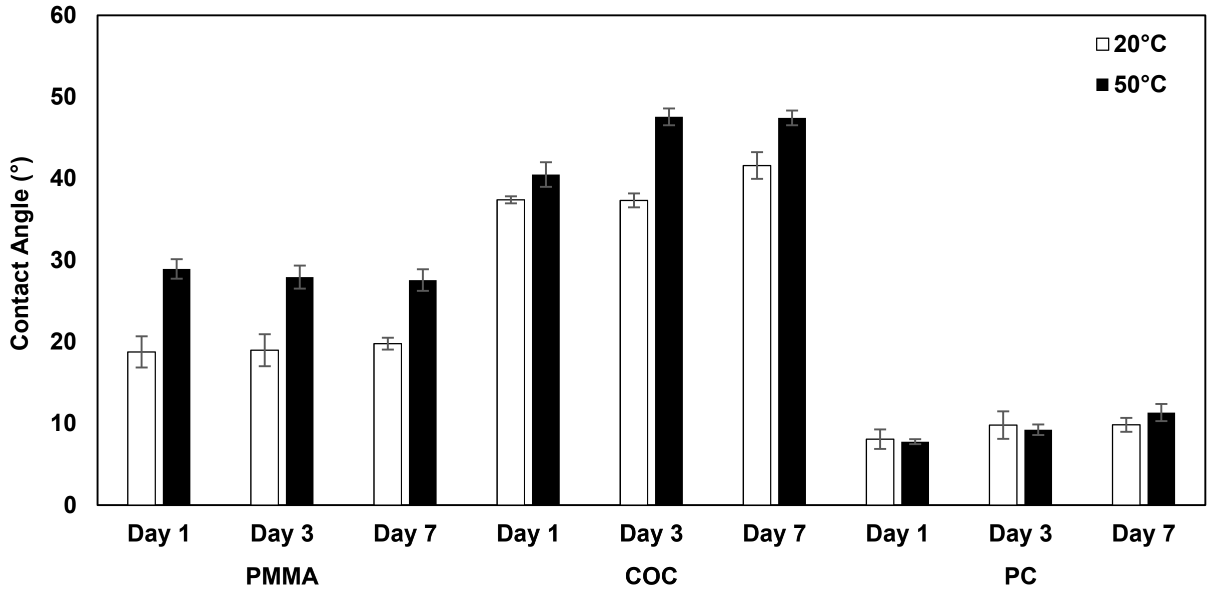

Fig. S3 Contact angles of UVO-treated (80 min) PMMA, COC and PC after 1, 3 and 7 days of storage in air at room temperature (20°C) and 50°C. Each bar represents the mean ± SD of five separate measurements.
